# A Case Study on Stratified Settlement and Rebound Characteristics due to Dewatering in Shanghai Subway Station

**DOI:** 10.1155/2013/213070

**Published:** 2013-05-19

**Authors:** Jianxiu Wang, Tianrong Huang, Dongchang Sui

**Affiliations:** ^1^Key Laboratory of Geotechnical and Underground Engineering of Ministry of Education, Tongji University, Shanghai 200092, China; ^2^College of Civil Engineering, Tongji University, Shanghai 200092, China

## Abstract

Based on the Yishan Metro Station Project of Shanghai Metro Line number 9, a centrifugal model test was conducted to investigate the behavior of stratified settlement and rebound (SSR) of Shanghai soft clay caused by dewatering in deep subway station pit. The soil model was composed of three layers, and the dewatering process was simulated by self-invention of decompressing devise. The results indicate that SSR occurs when the decompression was carried out, and only negative rebound was found in sandy clay, but both positive and negative rebound occurred in the silty clay, and the absolute value of rebound in sandy clay was larger than in silty clay, and the mechanism of SSR was discussed with mechanical sandwich model, and it was found that the load and cohesive force of different soils was the main source of different responses when decompressed.

## 1. Introduction

Located in the Yangtze Delta, Shanghai is a typical area of soft soil distribution. As one of the most dynamic economic center in the world, metro railway construction is now being developed on a large scale, and the location of metro stations are often in densely populated districts. The dewatering measures were often adopted in metro station constructions, which often cause soil layer compression, land subsidence, and foundation's deformation. Therefore, it is of vital importance to conduct research on it andfind out the corresponding measures to ensure the safety of construction and protection of the environment. For this reason, various researches have been conducted to investigate the relationship between land subsidence and water withdrawal by dewatering [1–11]. These studies discussed the effects of soil consolidation and land subsidence induced by dewatering, mostly in a perspective of mechanics as groundwater pumping leading to a decline in the groundwater level.

In the fields related to deformation of clay, Yuan et al. established a modified Cam-clay model with nonassociated flow rule considering S-D effect on deviatory plane and cohesion on meridian plane [12]. Yu et al. proposed a constitutive model to fully describe the mechanical behavior of the boom clay by combining the transversely isotropic elastic model and the modified Mohr-Coulomb criterion [13]. Taiebat et al. extended the SANICLAY model to include restructuration by conducting two distinct types of restructuration (isotropic and frictional) [14]. Liu and Carter described that the behavior of natural clay is proposed in a new four-dimensional space, consisting of the current stress state, stress history, the current voids ratio, and a measure of the current soil structure [15]. Cotecchia and Chandler suggested that the structure of clays may be distinguished simply as either sedimentation or postsedimentation, depending on whether gross yield in one-dimensional compression in void ratio-vertical effective stress plane occurs on the original sedimentation compression curve or to the right of this curve [16]. Mutman investigated the properties of bentonite clay stabilized by the burned olive waste and proposed a solution to the problem of the olive oil waste which are incriminated for a high quantity of pollution [17]. These works have made people gain more understanding about the characteristic of clay, but few were concerned about the special phenomenon (stratified settlement and rebound) in soft clay.

The stratified settlement means different settlement with different location in one layer, and the rebound includes positive and negative rebound, which was expansion and compression in the soil, respectively. The SSR was a brand new deformation behavior of soil; it was first discovered by Shanghai Tunnel Construction Corporation and reported by Wang et al. [18]; but this behavior had not been paid enough attention in engineering application. As the accelerating of the city's development, metro stations are now being conducted in more dense districts with considerable surrounding tall buildings, and the control of land subsidence become mores strictly. For example, the land subsidence needs to be controlled within 7 millimeters in Yishan metro station of number 3 Ming Zhu subway in Shanghai, PR, China [19]. Therefore, the research of stratified settlement and rebound is of great significance, for it affects the accurate calculation and control of land subsidence, which has also great potential in engineering application. In previous study [18], the SSR was mainly supported and explained by the field monitoring data, and no model tests have been conducted to do the relevant research, which prohibits the profound recognition of this phenomenon and potential application, so it is very necessary to conduct the centrifuge model test to research the mechanism of SSR.

This paper is to analyze the mechanism of SSR of Shanghai clay often found in the metro station construction by the centrifugal model test. For this purpose, the soil samples were obtained from the Yishan Metro Station Project of Shanghai Metro Line 9. Then, the model test was conducted by using the centrifuge of Tongji University and self-invention of decompressing devise, and the results are explicitly discussed. The relation between the distance from the center of pit and land subsidence is also discussed. Finally, the mechanism of SSR of Shanghai clay is analyzed based on a mechanical sandwich model. It brings useful reference to designers and is helpful to analyze and control ground subsidence in this situation.

## 2. Centrifugal Model Test

### 2.1. Engineering Background

**Figure 1:**
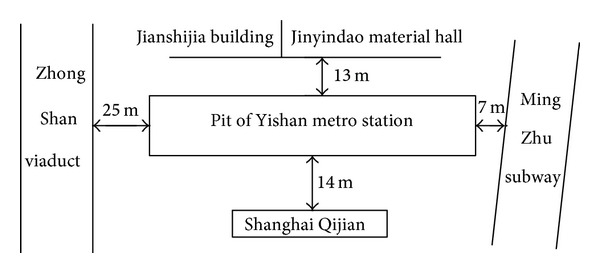
Layout of Yishan metro station.

Yishan Road station was located at Yishan Road with four storied island platform structure and the terminal station of Shanghai subway number 9. It extended from the west (West Zhongshan Road) to the east (Kaixuan Road) with a total distance of 297.40 m. The width of its standard part was 21.2 m, and the environmental conditions around Yishan Road station were very complex, and it required high level of environmental protection. The middle part of the south side of Yishan station was Shanghaiqijian decoration building with 17 storied concrete frame structure, which was 14 m away from the boundary of the enclosure protection of the pit; in the north of the station are Jianshijia building and Jinyindao material hall, which were 13 m away from the pit. In its east, there were Mingzhu viaduct and the Yishan Road station of Mingzhu subway. The minimum distance was 7 m between the pile cap of Mingzhu viaduct and the pit while that between the Yishan Road station of Mingzhu subway and the pit was 23 m; the west of the station was Zhong Shan viaduct with a minimum distance of 25 m between its center and the pit. All the buildings and roads had a strict requirement for settlement control which was mainly caused by dewatering of the confined aquifer. The location and neighboring building of Yishan Road station are presented in Figure 1. 

**Figure 2:**
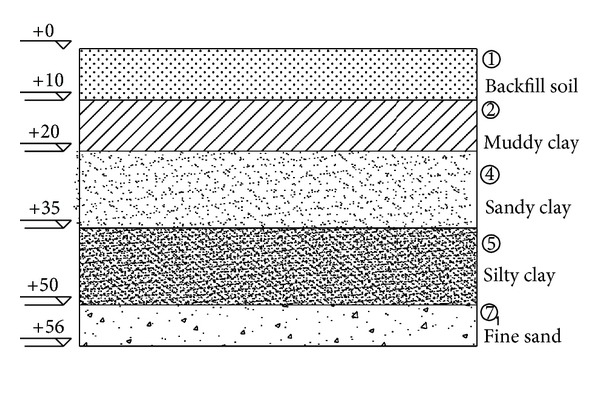
Geological section of Yishan metro station.

According to the geotechnical investigation report, the simplified geological section of Yishan Road station was shown in Figure 2.

### 2.2. The Geotechnical Centrifuge

**Figure 3:**
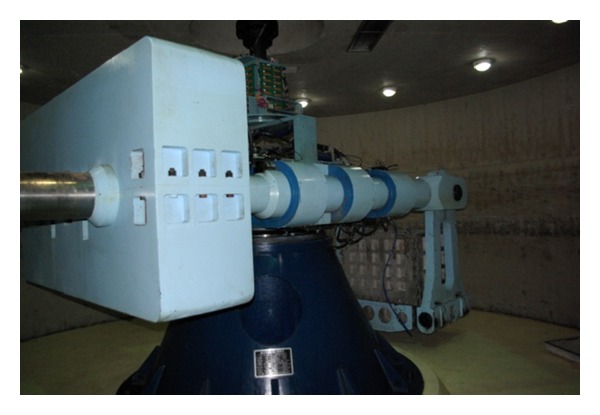
The geotechnical centrifuge of Tongji University.

This geotechnical centrifuge of Tongji University was shown in Figure 3, whose capacity is 150 g · t and maximum acceleration is 200 g, and it had been successfully applied to related research by a group of researchers in Tongji University [20–22].

### 2.3. Soil Properties and Preparation

Since the bearing stratum of metro stations is often located at the layer number 4 of sandy clay and layer number 5 of silty clay in Shanghai [23], this paper focus on this two kinds of soft clay in Shanghai. In this paper, in order to simulate the SSR, self-invention of decompressing devise was placed at the bottom of layer number 5 to simulate the decompression induced by dewatering in deep pit, and a topsoil layer of silty clay was placed on top of the two layers to stand for equal gravity. The properties of test soil are listed in Table 1.

**Figure 4:**
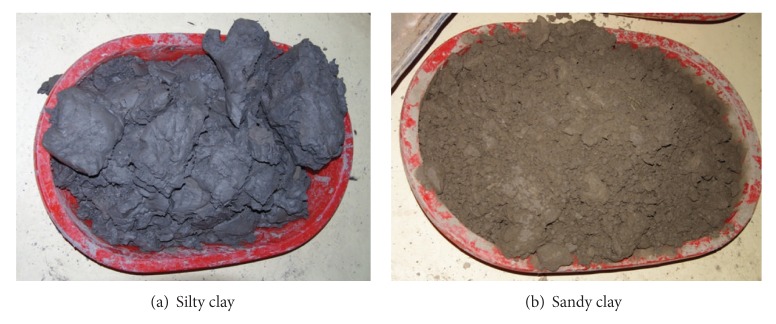
Pictures of soil samples.

The soil samples were demonstrated in Figure 4, and they were silty clay and sandy clay, respectively.

To minimize side friction, the wall of model was covered with a thin layer of smooth plastic membrane. The procedures of preparing soil layer were as follows:

1. the silt clay obtained from the site was used to construct the bottom silt clay (layer number 5), 150 mm in thickness;
2. layer No. 4 (150 mm in thickness) was constructed by the sandy clay obtained from the site;
3. last, 30-mm-thick topsoil layer was constructed on top of layer No. 4 and No. 5.

The model was consolidated at 150 g for about 3 h after soil layers were constructed. 

**Figure 5:**
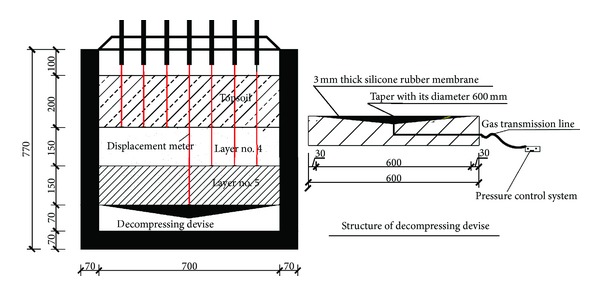
The distribution of stratum and decompressing devise in test model (unit: mm).

At the bottom of layer number 5, a self-invention of decompressing devise was adopted to simulate the decompression induced by dewatering in confined aquifer. It was composed by a plate of low-density polyethylene with 70 mm in thickness, 660 mm in length, and 660 mm in width, and a taper was on top of this plate with a 3 mm-thick silicone rubber membrane. The diameter of this taper was 600 mm, and the bottom of the taper was connected to the pressure control system by a gas transmission line. The decompression was simulated and controlled by the pressure control system. The soil layers and self-invention of decompressing devise were illustrated in Figure 5. 

### 2.4. Instrument and Test Procedures

**Figure 6:**
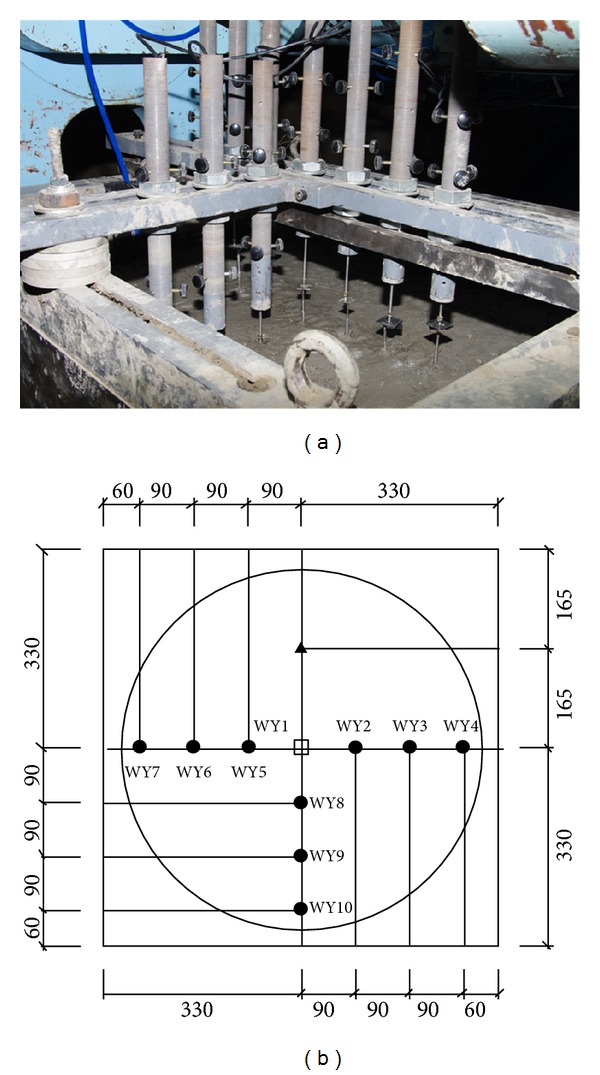
The distribution of displacement meters (unit: mm).

Confined to the size of test box, the model scale was taken as *n* = 20. The model box was 700 × 900 × 700 mm in width, length, and height, respectively. To measure the stratified settlement and rebound, nine displacement meters were placed as Figure 6 indicates.

In Figure 6, WY1 were set in the centre of the model and reached the top of the decompressing device, and the displacement meters WY8, WY9, and WY10 were placed on top of the topsoil, which was to measure the displacement on topsoil, and their distance from the centre of the model was 90 mm, 180 mm, and 270 mm, respectively. In the same way, WY5, WY6, and WY7 were located on top of layer number 4, and WY2, WY3, and WY4 were located on top of layer number 5. Therefore, WY8-WY5, which means the subtraction value of displacement WY8 and WY5, represents the settlement of layer number 4 with a distance of 90 mm from the model center, and WY9-WY6 and WY10-WY7 represented the settlement of layer number 4 with a distance of 180 mm and 270 mm from the model center, respectively. In the same case, WY5-WY2, WY6-WY3, and WY7-WY4 represented the stratified settlement of layer number 5 with a distance of 90 mm, 180 mm and 270 mm from the model center, respectively.

The centrifuge model test was conducted to study SSR of Shanghai clay. It has five accelerations, including 10 g, 20 g, 30 g, 40 g and 50 g. During the test, it needed 30 minutes to accelerate the centrifuge before it reached the stable stage. And the whole running time of the centrifuge was 2 h. 

## 3. Test Results and Analysis

To express concisely, the displacement on different layer surface in 30 g were first chosen to be discussed, then the SSR in layer No. 4 and No. 5 was investigated respectively, and then the SSR in different accelerations was also discussed. Finally the mechanism of SSR was investigated with a mechanical sandwich model.

### 3.1. Displacement on Different Layer Surface in 30 g

**Figure 7:**
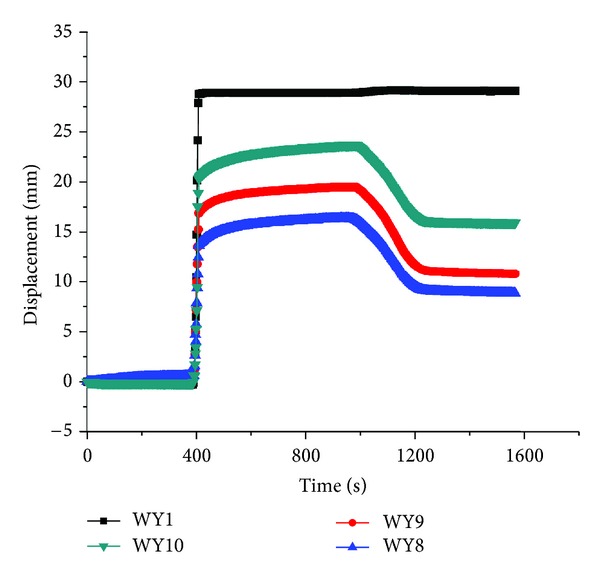
The displacement on top of topsoil.

**Figure 8:**
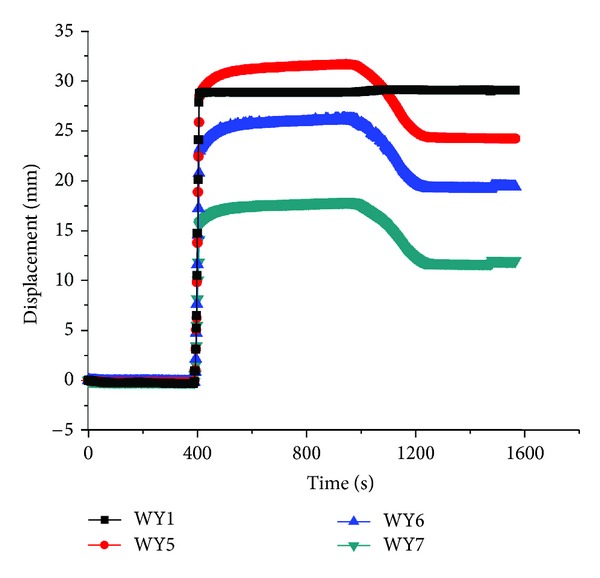
The displacement on top of layer number 4.

**Figure 9:**
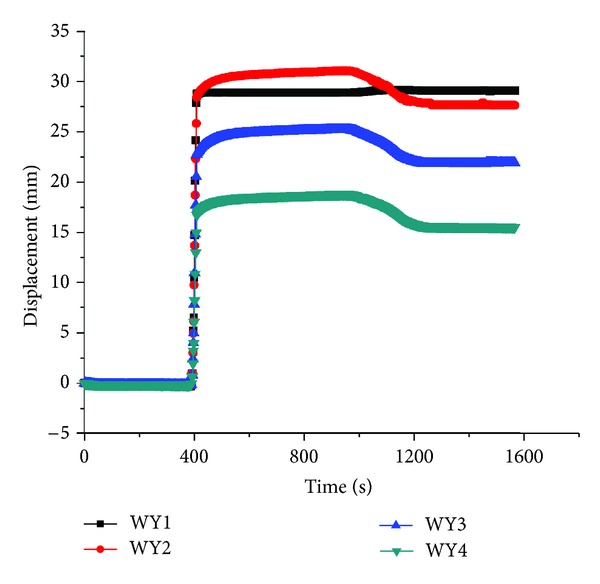
The displacement on top of layer number 5.

The displacement on each layer's surface was drawn in Figures 7, 8, and 9, respectively.

As indicated, there existed no displacements in the soil at the time of 400 seconds, during which the WY1 displacement meter remained zero and mean no decompression. However, the displacement of all layers came to a sudden change when displacement of WY1 increased to 28 mm, but the displacement of WY1 remained constant from 400 to 1000 seconds, which also mean the constant decompression at the bottom of layer No. 5, and then the displacement began to decrease with the unloading of centrifuge when the time exceeded 1000 seconds. This demonstrated that the decompression had a strong impact on soil deformation and surface movement. Comparing the three figures, it can also be found that the displacement on top of topsoil was the smallest among the three layers, which showed that the decompression exerted the greatest influence on the nearby layer number 5 but the least impact on the surface of topsoil. 

### 3.2. Stratified Settlement and Rebound

#### 3.2.1. Stratified Settlement and Rebound in 30 g

**Figure 10:**
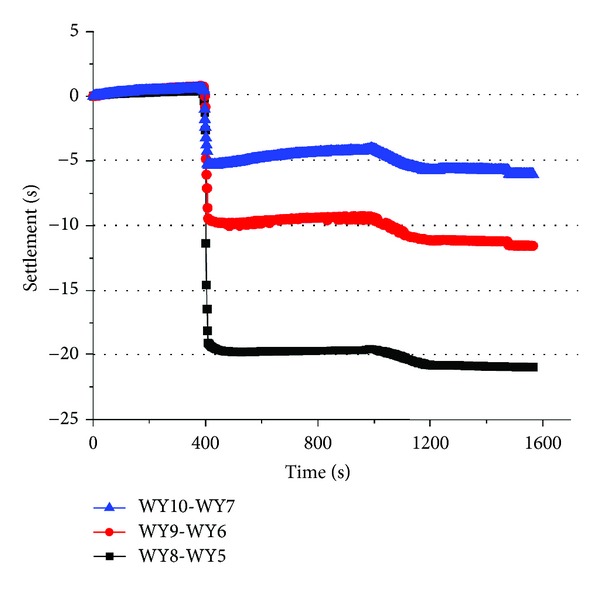
The settlement of layer number 4.

Based on the data in Figures 7 and 8, the settlement of layer number 4 was drawn in Figure 10.

As mentioned in Section 2.3, WY8-WY5, WY9-WY6, and WY10-WY7 represented the settlement of layer No. 4 with a distance of 90 mm, 180 mm, and 270 mm from model center, respectively. From Figure 8, it was found that the settlement of WY8-WY5, WY9-WY6, and WY10-WY7 remained zero before the time of 400 seconds. After the decompression began at 400 seconds, the settlement come to a sudden decrease but was of different value, respectively. The settlement of WY10-WY7 dropped from 0 mm to −5 mm, and the settlement of WY8-WY5 and WY9-WY6 decreased from 0 mm to −10.5 mm and −20.0 mm, respectively, so the settlement of layer number 4 was not unanimous with different location, which was called stratified settlement, and the absolute value of stratified settlement decreased with increasing distance from the model center. 

 On the other hand, the settlement after decompression also stands for the rebound occurred in the layer number 4. This rebound was negative and often called compression. And it was found that the rebound decreased with the distance from the model center too, and the rebound only occurred after decompression began in layer number 5, which showed that decompression was the main source of rebound. 

**Figure 11:**
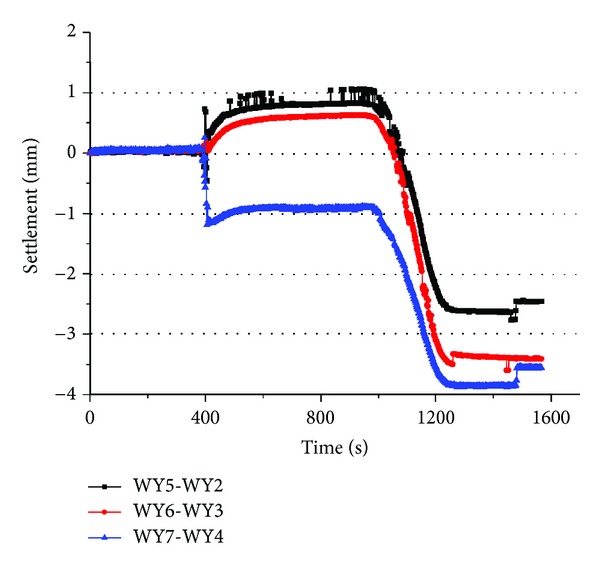
The settlement of layer number 5.

In the same way, according to the data of Figures 8 and 9, the settlement of layer number 5 was shown in Figure 11.

In Figure 11, WY5-WY2, WY6-WY3, and WY7-WY4 represent the settlement with a distance of 90 mm, 180 mm, and 270 mm in layer number 5 respectively. Similarly, the SSR was also found in layer number 5 with the decompression. However, there existed two kinds of rebound in layer number 5, positive rebound and negative rebound, which are also called expansion and compression, respectively; for the WY5-WY2 and WY6-WY3, the rebound was positive, so the soil was expanded when the decompression began, and it was also found that the value of expansion decreased with the increasing distance from model center, and for WY7-WY4, the rebound was negative which shows that the soil was compressed. In addition, comparing with Figure 7, we find that the absolute value of positive and negative rebound in layer number 5 was smaller than those in layer number 4.

In previous study [18], it was reported that the stratified settlement and rebound existed and decreased with the distance from the center of the pit when the dewatering measure was taken in the confined water. Therefore the centrifuge model test is feasible and keeping well confirmation with the field monitoring information.

#### 3.2.2. Stratified Settlement and Rebound in Different Acceleration

**Figure 12:**
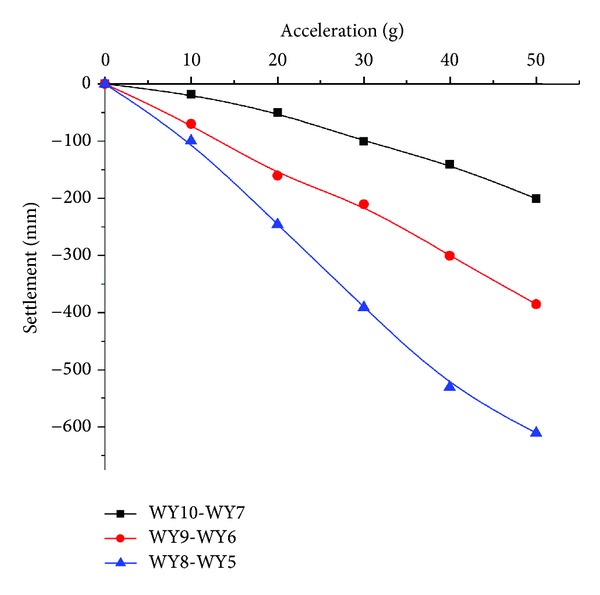
The settlement of layer number 4 in different accelerations.

**Figure 13:**
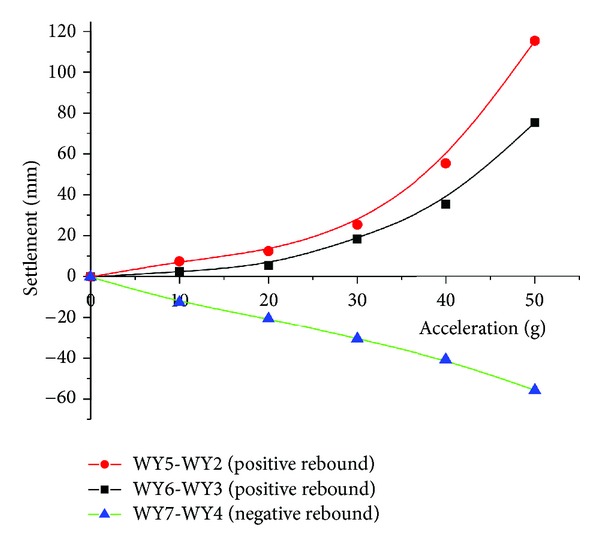
The settlement of layer number 5 in different accelerations.

According to the test scheme, the settlement of layer number 4 and number 5 in different acceleration was drawn in Figures 12 and 13 respectively (The value of SSR has multiplied with the scale 20 to make it more clearly).

As it was found in Figure 12 that, the settlement of layer number 4 increased with the acceleration; the bigger acceleration mean larger *t* settlement in the sandy clay, which may be interpreted that larger acceleration makes the soil more compact just like the soil's consolidation, and it was also found that the absolute value of settlement of layer number 4 also decreased with the increasing distance from model center for different series, which also means that the decompression exerted its impact on nearby zone in a more profound way. These results were similar to the field monitoring information, which demonstrates the validity of the SSR found in actually engineering. 

From Figure 13, the stratified settlement and positive or negative rebound were found in different accelerations for the silty clay of layer number 5. Even in different accelerations, the soil was expanded in the location with a distance of 90 mm; 180 mm from the model center; while it was compressed in the location with a distance of 270 mm. The deformation behavior of soil was varying with the distance from the model center, which was called the stratified settlement in Section 3.1. Moreover, the value of positive and negative rebound increased with the acceleration, and the bigger acceleration meant the larger positive rebound and smaller negative rebound. This may be explained that the higher acceleration means longer time of consolidation for the centrifuge which has a good amplification effect [20–22]. Therefore, the existence of the SSR was not temporarily, and it was the fresh new deformation behavior of silty clay. To make the land subsidence calculation more precisely, further investigation needs to be conducted in a quantitative way. 

### 3.3. The Mechanism of Stratified Settlement and Rebound

Generally speaking, the soil layers were located in a field of self-weight stress in normal condition. For the soil layers being of certain consolidation and stress, rebound would occur in the soil no matter the unloading was induced in the top or bottom of the layer, which was also proved by the rebound often observed at the bottom of the pit during deep pit excavation [24]. However, the soil was made by nature and always heterogeneous aggregate; its properties may differ with location and result in their different response of same loading. So the settlement varied with different distance from the model center in a specific layer. In addition, comparing with the properties index of layer number 4 and layer number 5, it is found that the cohesive force of layer number 4 is 14 kPa bigger than layer number 5. Because the cohesive force means the stronger internal bond and connection, the response of the two kinds of soil layers showed a different trend. In a whole, the property of the soil including the type and constitution of soil was the major effect leading to the stratified settlement.

**Figure 14:**
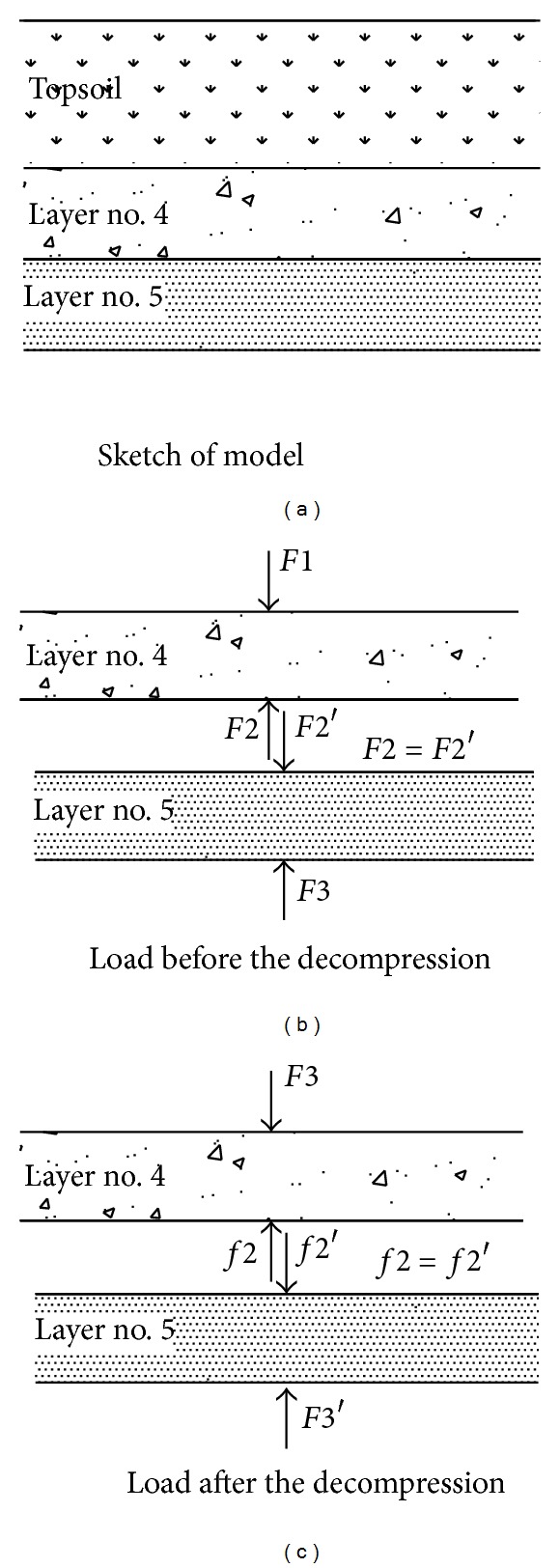
The sandwich model of soil.

In order to reveal this mechanism, the mechanical sandwich model was drawn in Figure 14.

As Figure 14 indicates, the load of each layer was different both before and after decompression. Layer number 4 and number 5 remained in a balance under the gravity of topsoil before the decompression. When the decompression began, the supporting force was decreasing at the bottom of layer number 5; it decreased from F3 to F3′ which lead to deformation occurred in layer number 5 before a new balance was attained. The bottom of layer number 5 began to move downward when decompression began. Meanwhile the top of layer number 5 also moved downward, but it moved at a smaller speed. And it was because the layer number 5 was silty clay with high cohesive force of 20 kPa. In the model center, the effect of the decompression was the biggest. All these made the settlement in layer number 5 become different with location, which was so called stratified settlement, and for WY5-WY2 and WY6-WY3, the displacement at the bottom was big enough to exceed on the top, so their rebound is positive which means expansion in the center nearby, but their value was small; it was 0.5 mm or 0.75 mm, respectively, and for the WY7-WY4, it was located at a distance of 270 mm from the test model. The effect of decompression was relatively smaller so the settlement of the top exceeded the bottom, so WY7-WY4 were negative rebounds which means compression at the zone far from the center. 

 For layer number 4, the gravity of topsoil remained the same on the top. As the decompression began at the bottom of layer number 5, layer number 4 also was influenced by the decreasing support, which led to an increasing load on top of layer number 4 correspondingly; its value was the subtraction of F1 to F2, which was smaller than the subtraction of F1 to F2. Moreover, the layer number 5 had higher cohesive force and some positive rebound the bottom of layer number 4 would also receive some of the expansion force. All these resulted in the negative rebound in layer number 4, which led to the compression in layer number 4. Similar to layer number 5, the rebound also decreased with the distance from the model center for the effect of decompression reduced gradually with the distance.

## 4. Conclusions

Through the centrifuge model test composed of three layers of soil and self-invention of decompressing devise, the decompression in pit dewatering was simulated successfully. The conclusion may be summarized as the following.

1. The centrifuge model test is feasible to simulate the SSR induced by decompression induced by dewatering in confined water during metro station construction.
2. The stratified settlement induced by decompression was found both in sandy clay and silty clay, and the absolute value of SSR decreased with increasing distance from the model center.
3. When decompression was conducted, only negative rebound was found in the sandy clay, but both positive and negative rebound were found in silty clay, and the absolute value of SSR in the sandy clay was bigger than the silty clay.
4. The stratified settlement and rebound in other acceleration was similar to the acceleration of 30 g; the absolute value of SSR in bigger acceleration was larger than the smaller acceleration, which showed larger acceleration made the soil more compact.
5. The mechanism of stratified settlement and rebound lies in the load in the soil layer and type of soil. The decompression exerted more profound impact on the bottom of layer number 5, and the different properties of sandy clay and silty clay also contributed to the different response of same decompression.
6. Owing to the model scale and the complexity of soil, current work is mainly to simulate the stratified settlement and rebound qualitatively; quantitative researches are needed to be conducted to make more profound recognition in future.

## Figures and Tables

**Table 1:** The properties of the soil samples.

Layer number	Name	Density (g/cm^3^)	Water content (%)	Porosity ratio	Cohesive force (kPa)	Internal friction angle (°)
Topsoil*	Silty clay	18.4	39.8	1.13	19	27.5
Layer number 4	Sandy clay	18.1	36.9	1.1	7	31
Layer number 5	Silty clay	18.4	39.8	1.13	20	27

*Means that layer topsoil is the layer with same gravity of all layers above layer number 4 and number 5.

## References

[1] Xue YQ, Zhang Y, Ye SJ, Wu JC, Li QF (2005). Land subsidence in China. *Environmental Geology*.

[2] Tang YQ, Cui ZD, Wang JX, Lu C, Yan XX (2008). Model test study of land subsidence caused by high-rise building group in Shanghai. *Bulletin of Engineering Geology and the Environment*.

[3] Tang YQ, Cui ZD, Wang JX, Yan LP, Yan XX (2008). Application of grey theory-based model to prediction of land subsidence due to engineering environment in Shanghai. *Environmental Geology*.

[4] Shi X, Wu J, Ye S (2008). Regional land subsidence simulation in Su-Xi-Chang area and Shanghai City, China. *Engineering Geology*.

[5] Wang J, Hu L, Wu L, Tang Y, Zhu Y, Yang P (2009). Hydraulic barrier function of the underground continuous concrete wall in the pit of subway station and its optimization. *Environmental Geology*.

[6] Shen SL, Xu YS (2011). Numerical evaluation on land subsidence induced by groundwater pumping in Shanghai. *Canadian Geotechnical Journal*.

[7] Xu YS, Ma L, Du Y-J, Shen S-L (2012). Analysis of urbanisation-induced land subsidence in Shanghai. *Natural Hazards*.

[8] Ding LY, Wu etc XG, Li H, Luo HB, Zhou Y (2011). Study on safety control for Wuhan metro construction in complex environments. *International Journal of Project Management*.

[9] Jiang S, Kong X, Ye H, Zhou N (2013). Groundwater dewatering optimization in the Shengli no. 1 open-pit coalmine, Inner Mongolia, China. *Environmental Earth Science*.

[10] Wang J, Fen B, Liu Y (2012). Controlling subsidence caused by de-watering in a deep foundation pit. *Bulletin of Engineering Geology and Environment*.

[11] Wang J, Feng B, Yu H, Guo T, Yang G, Tang J (2013). Numerical study of dewatering in a large deep foundation pit. *Environmental Earth Science*.

[12] Yuan K, Chen W, Yu H (2012). Modified Cam-clay model considering cohesion and S-D effect and its numerical implementation. *Chinese Journal of Rock Mechanics and Engineering*.

[13] Yu H, Chen W, Li X, Sillen X (2013). A transversely isotropic damage model for boom clay. *Rock Mechanics and Rock Engineering*.

[14] Taiebat M, Dafalias YF, Peek R (2010). A destructuration theory and its application to SANICLAY model. *International Journal for Numerical and Analytical Methods in Geomechanics*.

[15] Liu MD, Carter JP (2003). Volumetric deformation of natural clays. *International Journal of Geomechanics ASCE*.

[16] Cotecchia F, Chandler RJ (2000). A general framework for the mechanical behaviour of clays. *Geotechnique*.

[17] Mutman U (2013). Clay improvement with burned olive waste ash. *The Scientific World Journal*.

[18] Wang J, Wu L, Zhu Y, Tang Y, Yang P, Lou R (2009). Mechanism of dewatering-induced ground subsidence in deep subway station pit and calculation method. *Chinese Journal of Rock Mechanics and Engineering*.

[19] Tang YQ, Luan CQ, Wang JX, Zhu YF, Pan WQ (2008). Analysis of the effects of environments for dewatering in a metro station in shanghai. *Journal of Wuhan University of Technology*.

[20] Cui ZD, Tang YQ, Yan XX (2009). Centrifuge modeling of land subsidence caused by the high-rise building group in the soft soil area. *Environmental Earth Sciences*.

[21] Cui ZD, Tang YQ (2010). Land subsidence and pore structure of soils caused by the high-rise building group through centrifuge model test. *Engineering Geology*.

[22] Tang YQ, Ren X, Chen B, Song S, Wang JX, Yang P (2012). Study on land subsidence under different plot ratios through centrifuge model test in soft-soil territory. *Environmental Earth Sciences*.

[23] Gong SL (1998). The influence of urban construction in Shanghai to the land subsidence. *Chinese Journal of Geological Hazard Control*.

[24] Pan Y, Wu Z (2002). Experimental study on the resilience of pit unloading. *Chinese Journal of Geotechnical Engineering*.

